# Permissible Outcomes of Lobe-Specific Lymph Node Dissection for Elevated Carcinoembryonic Antigen in Non-Small Cell Lung Cancer

**DOI:** 10.3390/medicina57121365

**Published:** 2021-12-14

**Authors:** Hiroaki Kuroda, Junji Ichinose, Katsuhiro Masago, Yusuke Takahashi, Takeo Nakada, Masayuki Nakao, Sakae Okumura, Kohei Hashimoto, Yosuke Matsuura, Noriaki Sakakura, Hirokazu Matsushita, Mingyon Mun

**Affiliations:** 1 Department of Thoracic Surgery, Aichi Cancer Center Hospital, Nagoya 464-8681, Japan; y.takahashi@aichi-cc.jp (Y.T.); takeo521@hotmail.co.jp (T.N.); nsakakura@aichi-cc.jp (N.S.); 2 Department of Thoracic Surgical Oncology, Cancer Institute Hospital, Tokyo 135-8550, Japan; junji.ichinose@jfcr.or.jp (J.I.); masayuki.nakao@jfcr.or.jp (M.N.); sokumura@jfcr.or.jp (S.O.); kohei.hashimoto@jfcr.or.jp (K.H.); yosuke.matsuura@jfcr.or.jp (Y.M.); mingyon.mun@jfcr.or.jp (M.M.); 3 Department of Pathology and Molecular Diagnostics, Aichi Cancer Center, Nagoya 464-0021, Japan; masago@aichi-cc.jp; 4 Division of Translational Oncoimmunology, Aichi Cancer Center Research Institute, Nagoya 464-0021, Japan; h.matsushita@aichi-cc.jp

**Keywords:** non-small cell lung cancer, lobe-specific lymph node dissection, systemic lymph node dissection, carcinoembryonic antigen, lobectomy

## Abstract

*Background and Objectives:* Lobe-specific nodal dissection (L-SND) is currently acceptable for the dissection of early-stage non-small cell lung cancer (NSCLC) but not for cancers of more advanced clinical stages. We aimed to assess the efficacy of L-SND, compared to systemic nodal dissection (SND). *Materials and Methods*: We retrospectively collected the clinical data of patients with carcinoembryonic antigen (CEA) abnormality who underwent complete resection of NSCLC via lobectomy or more in addition to either SND or L-SND at two cancer-specific institutions from January 2006 to December 2017. *Results*: A total of 799 patients, including 265 patients who underwent SND and 534 patients who underwent L-SND, were included. On multivariate analysis, thoracotomy, more than lobectomy, cN1-2, advanced pathological stage, adjuvant treatment, and *EGFR* or *ALK* were strongly associated with SND. No significant differences were found in overall survival, disease-free survival, and overtime survival after propensity adjustment (*p* = 0.09, *p* = 0.11, and *p* = 0.50, respectively). There were no significant differences in local (*p* = 0.16), regional (*p* = 0.72), or distant (*p* = 0.39) tumor recurrence between the two groups. *Conclusions*: SND did not improve the prognosis of NSCLC patients with CEA abnormality. Complete pulmonary resection via L-SND seems useful for NSCLC patients with CEA abnormality.

## 1. Introduction

Precise mediastinal lymph node staging is an essential strategy in the treatment of resectable non-small cell lung cancer (NSCLC). The standard surgical procedure comprises lobectomy or more with systemic nodal dissection (SND) [1,2]. However, lobe-specific nodal dissection (L-SND) was recently considered as an alternative to SND for the dissection of early-staged NSCLCs. Several authors have reported lobe-specific lymph node (LN) spread patterns [3]. However, SND is considered an important option, based on the following: (a) subcarinal or upper mediastinal metastases are rarely identified in upper or lower lobe NSCLC [4] and (b). SND and L-SND have similar effects on oncological prognosis but L-SND offers a significant advantage in reducing postoperative complications, especially in patients with cN0 or stage I NSCLC [3]. However, there are still some clinical studies with adverse results, and few reports have focused on L-SND [5].

According to the guidelines of the European Society of Thoracic Surgeons, SND is recommended in all cases to ensure complete resection, and L-SND is acceptable for peripheral squamous T1 tumors if hilar and interlobar nodes are negative on frozen section studies [6]. Besides, an anatomic pulmonary resection is preferred for most patients with NSCLC for the investigation of mediastinum [6]. The National Comprehensive Cancer Network recommends a minimum of three N2 station samples or complete lymph node dissection [7]. If nodal upstaging is detected after mediastinal LN dissection, patients with stage II tumors or tumors of higher stages should be referred for oncological evaluation. SND, or at least L-SND, can provide reliable staging in NSCLC patients, which can indicate the need for adjuvant therapy in some patients [8]. There may be a potential survival benefit for patients in whom NSCLCs were upstaged by the surgical identification of occult LN metastases.

Many factors, including thoracoscopic surgery [9], carcinoembryonic antigen (CEA) [10], standard uptake value on 18-fluorodeoxyglucose positron emission tomography [11], large tumor size [12], and lymph/vascular and/or pleural invasion [13] have been reported to be associated with occult hilar or mediastinal LN metastases, even in clinically diagnosed early-staged NSCLC. A representative tumor marker, such as CEA, is clinically used, but only a limited number of NSCLC patients significantly benefit [14]. Nasralla et al. reported that identifying patients with a poor prognosis, based on their high CEA level, may enable a more tailored approach in post-resection surveillance and patient counselling [15].

We previously reported that both imaging features on computed tomography (solid component or large mediastinal size) and high CEA level were highly correlated with the degree of tumor aggressiveness, enabling more accurate preoperative and intraoperative staging in early-staged NSCLC [11,13]. However, it has not been established whether L-SND is appropriate for NSCLC patients with CEA abnormality. The aim of this study was to investigate the patient selection bias between L-SND and SND at two cancer-specific institutions and compare the prognosis of patients after propensity adjustment. The two participating institutions employed the same strategy for nodal dissection, to reduce technical error in assessing the efficacy of intraoperative LN evaluation.

## 2. Materials and Methods

### 2.1. Study Population

**Figure 1:**
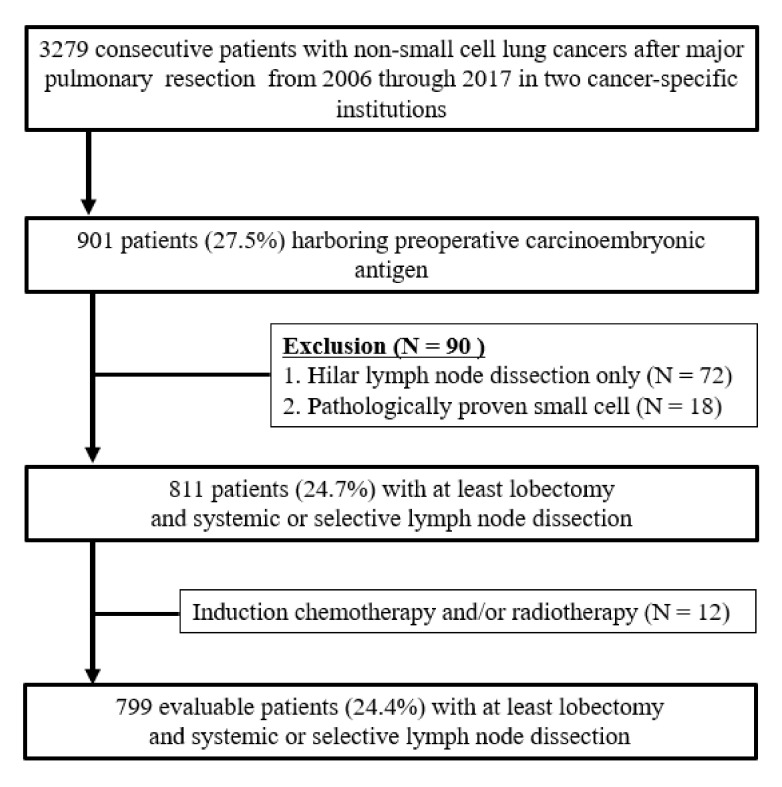
Flow chart of patient selection process.

This retrospective study was approved by the institutional review board of Aichi Cancer Center Hospital (2020-1-613), and the study protocol was performed in adherence with the Declaration of Helsinki. The requirement of informed patient consent was waived because of the retrospective nature of this study. Between January 2006 and December 2017, 3279 consecutive NSCLC patients underwent lobectomy or more with systemic lymph node dissection at two cancer-specific institutions. Among them, 901 patients (27.5%) were pointed out abnormal serum levels of CEA, preoperatively. The following exclusion criteria were applied: (1) patients with hilar lymph node dissection alone, (2) patients with a final diagnosis of small cell lung cancer, (3) patients who were receiving induction chemotherapy with or without radiotherapy, and (4) patients who had undergone sublobar resection. Finally, as shown in Figure 1, 799 patients (24.4%) were eligible for this cohort.

The medical records of the patients were reviewed, and the following information was collected: age, sex, smoking history, preoperative serum CEA level, clinical N status, tumor histology, surgical approach, procedures, adjuvant treatments, pathological stage, and mutation status, including whether epidermal growth factor (*EGFR*) or anaplastic lymphoma kinase (*ALK*) were present. The 8th edition of the Union for International Cancer Control (UICC)/American Joint Committee on Cancer (AJCC) TNM staging criteria were used for pathological staging of tumors [16].

### 2.2. Surgical Procedure and Outcome Measures

**Figure 2:**
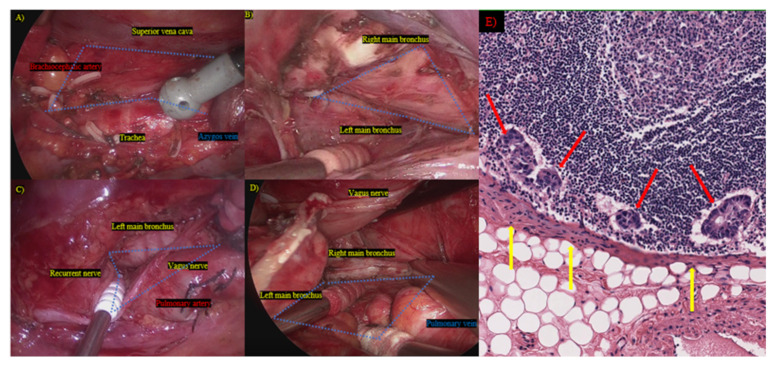
Mediastinal lymph node dissection and pathological findings. (**A**) Right upper mediastinum; (**B**) right bifurcation; (**C**) left upper mediastinum; (**D**) left bifurcation; blue area: mediastinal dissected region; (**E**) cancer cells disseminate from metastasized lymph nodes through the sheath of the lymph nodes; red arrow: metastasized lymph node; yellow arrow: disseminated cancer cells.

All patients underwent lobectomy or more. Thoracic surgeons in the two institutions applied the same principles for mediastinal lymph node dissection, using both thoracotomy and thoracoscopic surgery (Figure 2A–D).

We measured overall survival (OS) from the date of surgical resection to the date of death due to any cause or the date of the last follow-up. Disease-free survival (DFS) was defined as the period between the date of pulmonary resection and the date of recurrence, and in the absence of cancer-related death.

### 2.3. Statistical Analyses

All the computations were performed using standard software (SPSS version 25.0; SPSS Inc., Chicago, IL, USA). The comparisons between the two groups were performed using the Mann–Whitney *U*-tests. Baseline variables that were considered clinically relevant or that revealed a significant difference in univariate analysis were entered into the multivariate model. Propensity adjustment was defined as conditional probability calculated using preoperative covariates. The Kaplan–Meier method was used to analyze survival rates in patient subsets; between-group differences in survival were assessed using the log-rank test. Potential correlates of survival were subjected to univariate and multivariate analyses using the Cox proportional hazards regression model. Hazard ratios (HRs) and median survival rates are presented as 95% confidence intervals (CIs). Statistical significance was set at *p* < 0.05.

## 3. Results

### 3.1. Unadjusted Baseline Characteristics

The study cohort included 799 patients (485 (60.7%]) men and 314 (39.3%) women) with NSCLCs and CEA abnormality (median: 8.5; interquartile range (IQR): 6.2–15.5). The patient flow algorithm is illustrated in Figure 1. Patient characteristics before and after propensity adjustment are summarized in Table 1 and Table 2, respectively. The range of mediastinal lymph node dissection in the two cancer-specific institutions, including en bloc lymph node dissection with the exposure of remnant surrounding structures, is depicted in Figure 1. This method was useful and feasible because cancer can metastasize through the sheath of a lymph node (Figure 2E).

Before PS adjustment, compared with SND patients, L-SND patients were older (*p* < 0.01) and the tumor stage in a majority of the patients was cN0 (*p* < 0.01). Regarding surgical approach, the proportion of patients who underwent thoracoscopy was higher in the L-SND group than in the SND group (*p* < 0.01), and lobectomy was performed more frequently in the L-SND group than in the SND group (*p* < 0.01). Cancer of more advanced pathological stage was diagnosed in a higher proportion of patients in the SND group compared to the L-SND group (*p* < 0.01). Therefore, compared to those who underwent L-SND, more patients who underwent SND received adjuvant chemotherapy with or without radiotherapy (*p* < 0.01). The 30 day and 90 day mortalities in the L-SND group were 0.38% and 0.75%, respectively, while those in the SND group were 0.55% and 0.74%, respectively. Significant differences were not found between two groups in 30 day and 90 day mortality (*p* = 0.73 and *p* = 0.99, respectively).

### 3.2. Surgical Procedure, Approach, and Hazard Ratios for Overall Survival in Patients with CEA Abnormality

**Figure 3:**
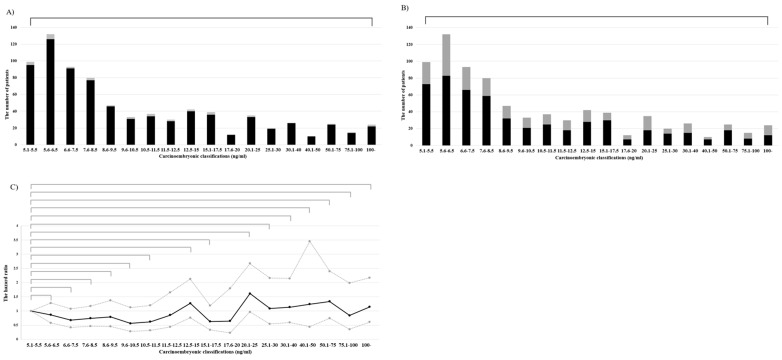
Surgical procedures and outcomes. (**A**) Distribution of lobectomy. Black: lobectomy; gray: more than lobectomy. (**B**) Distribution of lymph node dissection. Black: lobe-specific lymph node dissection; gray: systemic lymph node dissection. (**C)** Black line: Hazard ratio; gray line: 95% confidential limits.

No significant differences were found in the proportion of lobectomy at any CEA level (*p* = 0.95) (Figure 3A), nor for the proportion of L-SND (*p* = 0.65) (Figure 3B). When comparing the HRs of the lowest CEA value (5.1–5.5 ng/mL), the HRs for OS increased gradually as the CEA increased. However, by comparison, OS at any CEA value did not differ significantly (*p* = 0.07–0.81) (Figure 3C). Therefore, the CEA subset boundary could not be established.

### 3.3. Clinicopathological Factors Associated with Systemic Lymph Node Dissection

The multivariate analysis, which incorporated the results of univariate analysis, showed that thoracotomy, bilobectomy or pneumonectomy, cN1-2, advanced pathological stage, adjuvant chemotherapy with/without radiotherapy, and EGFR or ALK were strongly associated with SND (Table 3).

### 3.4. Surgical Outcomes

The median follow-up duration was 61.9 months (IQR: 40.0–85.6); in total, 328 (41.1%) patients died during this period. Cox multivariate regression analyses, which incorporated the results of univariate analysis, revealed that age, thoracotomy, SND, and advanced pathological stage were independent factors for an unfavorable prognosis (Table 4).

**Figure 4:**
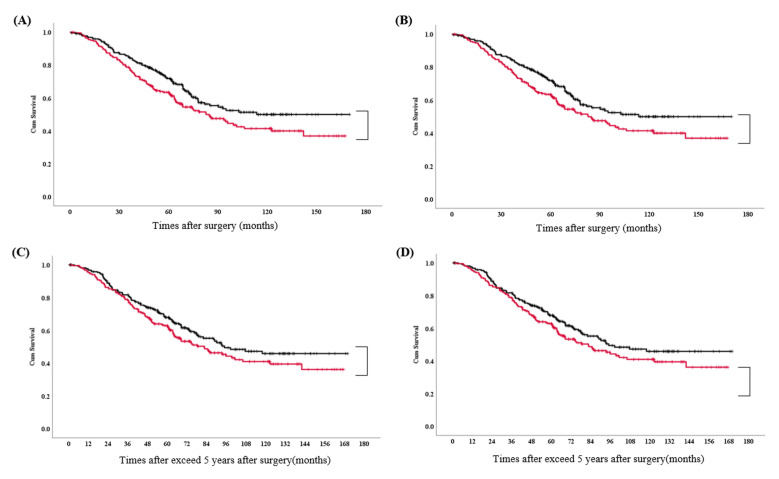
Kaplan–Meier curves. Overall survival curve (**A**) and disease-free survival curve (**B**) stratified by the degree of mediastinal lymph node dissection. Overall survival curves after 5 years of follow-up, before (**C**) and after (**D**) propensity adjustment. Black line: lobe-specific lymph node dissection; red line: systemic lymph node dissection.

Figure 4A illustrates the OS curves after propensity adjustment, according to the degree of LN dissection. The two- and five-year OS rates for L-SND and SND were 86.2% and 69.0%, and 86.3% and 60.2%, respectively. A significant difference was not found between the two (*p* = 0.09).

Figure 4B illustrates DFS curves after propensity adjustment, according to the degree of LN dissection. The two- and five-year DFS rates for L-SND and SND were 58.7% and 44.0%, and 50.2% and 35.6%, respectively. A significant difference was not found between the two (*p* = 0.11).

The survival benefit in over time of exceed 5 years for L-SND vs. SND gradually disappeared in both unadjusted and adjusted PS (*p* = 0.21 and *p* = 0.49, respectively) (Figure 4C,D).

There were no significant differences after propensity adjustment for local (*p* = 0.16), regional (*p* = 0.72), and distant (*p* = 0.39) tumor recurrence.

## 4. Discussion

This study demonstrated that SND offers no superiority over L-SND for surgical outcomes in patients with preoperative CEA abnormality before and after 5 years. Thoracic surgeons tend to perform SND to obtain precise pathological information from the thorough mediastinal nodal dissection. In addition, we conducted a retrospective analysis of clinicopathological variables that may influence patient prognosis during the same period. The results of this study can be summarized as follows: (1) our surgeons were able to perform SND in patients with CEA abnormality in terms of thoracotomy, bilobectomy or pneumonectomy, cN1-2, pathological advanced stages, adjuvant chemotherapy ± radiotherapy, and *EGFR* or *ALK*; (2) SND was equivalent to L-SND in OS and DFS, and (c) There was no significant difference in local (*p* = 0.16), regional (*p* = 0.72), and distant (*p* = 0.39) tumor recurrence. The superiority of SND to L-SND in prognosis was not verified in this study; however, our results cannot be related to patients with cancers of advanced stages. In addition, further randomized clinical trials are required.

Intraoperative SND is currently established as the standard procedure for resectable NSCLC, which is desirable in clinical practical applications in international guidelines [6,7]. SND can be used for precise N staging and for detecting occult lymph nodes. In our previous report, overall nodal upstaging was identified in 19 (7.8%) of 243 NSCLCs of clinical stage N0 using preoperative computed tomography (CT) and positron emission tomography (PET). These 19 cases included 10 (4.1%) mediastinal LN upstaging (cN0→pN2) and 9 (3.7%) hilar LN upstaging (cN0→pN1) [17]. Therefore, it serves as an index of further adjuvant chemotherapy for patients with R0 resected NSCLC or additional postoperative radiation therapy for those with non-R0. A previous review suggested that even if underlying radical differences in lung tumor biology contribute to this variation, there are also possible disparities related to the aggressiveness of preoperative and intraoperative nodal assessment that led to differences in outcomes [18]. In addition, the extent of the lymph node, which harbors biological heterogeneity, including the lymphatic flow in pN1-2 NSCLCs, might produce more accurate staging without an increase in morbidity and mortality [19]. SND is well known as the best way to acquire a correct nodal stages and individual lymphatic flows [20].

The lymphatic drainage route of NSCLC has been shown to depend on cancer location, flowing from inside to outside, near to distant, and from the intrapulmonary lobe to the hilar to the mediastinal lymph node [21]. However, some authors argue that cancer location is not a predictor of lymph node drainage pathway, and intraoperative SND is robustly preferred to L-SND, even for stage IA cancers [20]. However, several authors have reported that L-SND and SND have similar effects in the dissection of stage IA NSCLC for the following reasons: (a) equivalent survival outcomes were detected between L-SND and SND, (b) rare frequency of deviant lymph node metastases from lobe-specific lymphatic pathways, and (c) dismal prognosis in cases of deviant metastases [4,22]. Recently, Zhao et al. reported that L-SND had similar efficacy to SND in terms of survival, recurrence, lymph node dissection, and perioperative recovery in patients with clinical IA solid-dominant NSCLC, as well as significant advantages in reducing operative complications (after propensity matching) [3]. Although further investigation is needed, our results add to the literature because they indicate that L-SND might be more suitable than SND, even in patients with CEA abnormality.

Many recent studies that investigated CEA abnormality focused more on risk factors for hilar or mediastinal LN metastases in stage IA NSCLCs [23]. In our previous report, elevated CEA level was a borderline significant factor in skip N2 metastases in stage IA NSCLCs (*p* = 0.06) [24]. In the present study, CEA was also significantly higher in stage III cancers than in stage I to II cancers (*p* < 0.01, median (IQR): 10.4 ((6.6–23.0) vs. 8.0 (6.0–12.9)). A recent meta-analysis revealed that higher serum CEA levels were associated with advanced cancer stages and poor prognosis [25]. However, in this study, each layered CEA did not detect a significant difference in prognosis compared to the lowest group (5.1 to 5.5 ng/mL) (Figure 3C). A considerable reason why the difference in prognosis was reduced is the use of postoperative adjuvant treatment. Adjuvant treatment offers promising survival benefits for patients with stage III NSCLC compared to receiving surgery alone [26]. In this study, the five-year OS was similar, with or without adjuvant treatment, in patients with stage I or II NSCLC, while the five-year OS in patients with stage III NSCLC was significantly higher with adjuvant treatment (Supplementary Materials Figure S1a,b). In addition, CEA levels were significantly higher in patients who received adjuvant treatment than in those with stage I to II cancer.

This study featured several limitations. First, given the retrospective nature of the study, various patient selection biases were noted. However, this study was conducted at two cancer-specific institutions. We believe that the results of this study are reliable. This is because we used the same concept of LN dissection, which involves exposing the remnant structures and performing en bloc dissection, including the resection of fat and capsulized LN (Figure 2A,B). Hence, propensity adjustment using a relatively large-scale database may enable a more reliable and comprehensive analysis of surgical outcomes. Second, the proportion of thoracoscopic surgery was ≥30% from 2013 but did not exceed 50% in NSCLC patients with CEA abnormality. However, the five-year OS before propensity adjustment was significantly better in the L-SND (*n* = 407) group than in the SND (*n* = 240) group, among those who underwent thoracotomy, whereas those in L-SND (*n* = 125) and SND (*n* = 25) (Supplementary Materials Figure S2a,b). These data suggest that less invasiveness might affect prognosis, even in SND, but patient selection bias and a small sample size were both limitations of the present study. Recently, the demand for thoracoscopic surgery has increased, and the accumulation of data for future study is necessary.

## 5. Conclusions

Our study outcomes suggest that L-SND and SND offer similar efficacy in terms of short- or long-term prognosis and the recurrence of NSCLC with CEA abnormality. Therefore, complete resection via L-SND may be more applicable for NSCLC with CEA abnormality, but it may not be suitable for advanced-stage NSCLCs. Future randomized trials are required.

## Figures and Tables

**Table 1:** Characteristics of the tissues used for genomic tests.

Characteristics	Systemic Lymph Node Dissection	Lobe-Specific Lymph Node Dissection	*p*
	*n* = 265	*n* = 534	
Age (years old),	66	68	<0.01
median (IQR)	(63–74)	(60–71)	
Sex, male (%)	157 (59.2%)	328 (61.4%)	0.55
Smoking history,	30.0	33.9	0.68
pack-year (median, IQR)	(0–51.0)	(0–50.0)	
Carcinoembryonic antigen (ng/mL)	9.4	8.3	0.11
median, IQR	(6.2–18.4)	(6.3–14.5)	
Clinical stage *n* (number, %)			<0.01
cN0	164 (61.9%)	407 (76.2%)	
cN1-2	101 (38.1%)	127 (23.8%)	
Histology (number, %)			0.29
Adenocarcinoma	183 (69.1%)	388 (72.7%)	
Squamous	48 (18.1%)	86 (16.1%)	
Others	34 (12.8%)	60 (11.2%)	
Surgical approach (number, %)			<0.01
Thoracoscopy	25 (9.4%)	127 (23.8%)	
Thoracotomy	240 (90.6%)	407 (76.2%)	
Type of procedures (number, %)			<0.01
Lobectomy	235 (88.7%)	529 (99.1%)	
Pneumonectomy/Bilobectomy	30 (11.3%)	5 (0.9%)	
Adjuvant chemotherapy ± Radiotherapy			<0.01
(yes, %)	139 (52.5%)	117 (21.9%)	
Pathological stage			<0.01
IA1/IA2/IA3/IB	5/19/16/35	13/79/47/133	
IIA/IIB	8/60	28/90	
IIIA/IIIB	101/21	115/19	
Mutation status			<0.01
*EGFR* or *ALK* positive	106	148	
No mutations or uninformative	159	386	

IQR, interquartile range; *EGFR*, epidermal growth factor receptor; *ALK*, anaplastic lymphoma kinase.

**Table 2:** Clinicopathological characteristics after propensity adjustment.

Characteristics	Systemic Nodal Dissection	Lobe-Specific Nodal Dissection	*p*
	*n* = 219	*n* = 219	
Age (years old),	67	66	0.69
Median, IQR	(61–72)	(61–72)	
Sex, male (%)	131 (59.8%)	132 (60.3%)	0.92
Smoking history,	30.0	35.0	0.64
pack-year (median, IQR)	(0–52.0)	(0–51.0)	
Carcinoembryonic antigen (ng/mL)	8.9	8.3	0.82
median, IQR	(6.2–19.2)	(6.5–17.0)	
Clinical stage *n* (number, %)			0.83
cN0	154 (70.3%)	156 (71.2%)	
cN1-2	65 (29.7%)	63 (28.8%)	
Histology (number, %)			0.36
Adenocarcinoma	153 (69.9%)	162 (74.0%)	
Squamous	39 (17.8%)	33 (15.1%)	
Others	27 (12.3%)	24 (10.9%)	
Surgical approach (number, %)			0.64
Thoracoscopy	25 (9.4%)	22 (10.0%)	
Thoracotomy	194 (90.6%)	197 (90.0%)	
Type of procedure (number, %)			0.76
Lobectomy	213 (97.3%)	214 (97.7%)	
Pneumonectomy/bilobectomy	6 (2.7%)	5 (2.3%)	
Adjuvant chemotherapy ± Radiotherapy (yes, %)	88 (40.2%)	86 (39.3%)	0.85
Pathological stage			0.29
IA1/IA2/IA3/IB	5/19/14/34	3/21/11/46	
IIA/IIB	8/49	12/47	
IIIA/IIIB	75/15	69/10	
Mutation status			0.77
*EGFR* or *ALK* positive	83 (37.9%)	86 (39.3%)	
No mutations or uninformative	136 (62.1%)	133 (60.7%)	

IQR, interquartile range; *EGFR*, epidermal growth factor receptor; *ALK*, anaplastic lymphoma kinase.

**Table 3:** Univariate and multivariate analyses for systemic lymph node dissection.

Variables	Univariate	Multivariate	
	*p*	Hazard Ratio (95% CI)	*p*
Patient characteristics			
Age	<0.01 *	1.02 (1.01–1.04)	0.17
Male	0.55		
Pack-year	0.77		
Carcinoembryonic antigen			
Level	0.76		
Clinical N stage			
N1-2	<0.01 *	0.53 (0.36–0.79)	<0.01 *
Histology			
Adenocarcinoma or SQCC	0.32		
Procedures			
More than lobectomy	<0.01 *	0.11 (0.04–0.30)	<0.01 *
Approach			
Thoracotomy	<0.01 *	1.87 (1.13–3.05)	0.01 *
Adjuvant			
Chemotherapy and/or radiotherapy	<0.01 *	0.60 (0.42–0.86)	<0.01 *
Pathological stage			
More advanced	<0.01 *	1.14 (1.04–1.26)	<0.01 *
Mutation status			
*EGFR* or *ALK* positive	<0.01 *	0.53 (0.38–0.75)	<0.01 *

SQCC, squamous cell carcinoma; *EGFR*, epidermal growth factor receptor; *ALK*, anaplastic lymphoma kinase; CI, confidence index. *p* < 0.05, significant *.

**Table 4:** Univariate and multivariate analyses for overall survival.

Variables	Univariate	Multivariate	
	*p*	Hazard Ratio (95%CI)	*p*
Patient characteristics			
Age	<0.01 *	1.04 (1.03–1.05)	<0.01 *
Female (vs. male)	<0.01 *	0.82 (0.62–1.09)	0.16
Pack-year	<0.01 *	1.04 (1.03–1.05)	0.14
Carcinoembryonic antigen			
Value	0.58		
Clinical N stage			
N0 (vs. cN1-2)	<0.01 *	0.53 (0.36–0.79)	0.07
Histology			
Adenocarcinoma or SQCC	<0.01 *		0.48
Procedures			
More than lobectomy	<0.01 *	0.92 (0.58–1.48)	0.74
Approach			
Thoracotomy (vs. Thoracoscopy)	<0.01 *	1.56 (1.03–2.37)	0.04 *
Lymph node dissection			
L-SND (vs. SND)	<0.01	0.78 (0.61–0.99)	0.04 *
Adjuvant			
Chemotherapy and/or radiotherapy (vs. No)	0.37		
Pathological stage			
More advanced	<0.01 *	1.32 (1.22–1.43)	<0.01 *
Mutation status			
*EGFR* or *ALK* positive (vs. negative)	<0.01 *	1.20 (0.90–1.60)	0.22

SQCC, squamous cell carcinoma; L-SND, lobe-specific nodal dissection; SND, systemic nodal dissection; *EGFR*, epidermal growth factor receptor; *ALK*, anaplastic lymphoma kinase; CI, confidence interval. *p* < 0.05, significant *.

## Data Availability

In this section, data are available upon reasonable request from the corresponding author.

## Supplementary Materials

The following are available online at https://www.mdpi.com/article/10.3390/medicina57121365/s1, Figure S1: Kaplan-Meier curves; Figure S2: Surgical procedures and outcomes.
