# Supplementary material for: Permissible Outcomes of Lobe-Specific Lymph Node Dissection for Elevated Carcinoembryonic Antigen in Non-Small Cell Lung Cancer
Source: Medicina (Kaunas). 2021 Dec 14;57(12):1365. doi: 10.3390/medicina57121365 (PMC8709178; doi:10.3390/medicina57121365)
Supplement: Supplementary file 1 [file medicina-57-01365-s001.zip › medicina-1483937 supplementary.pdf]

Supplemental S1

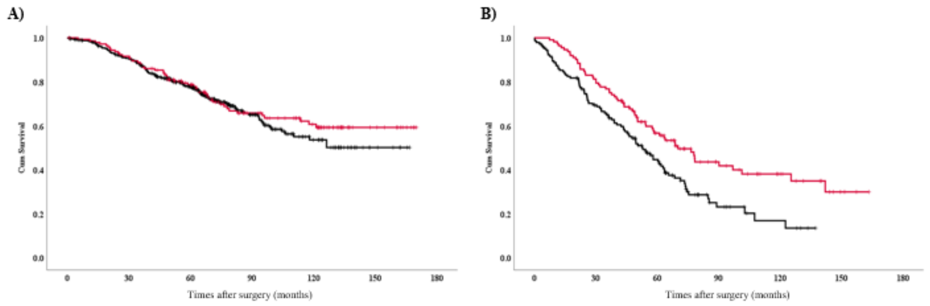

Figure S1. Kaplan-Meier curves. Overall survival curves stratified by adjuvant chemotherapy in stage I to II non-small cell lung cancers (A) and stage III cancers (B). Black line: lobe-specific lymph node dissection; Red line: systemic lymph node dissection.

Supplemental S2

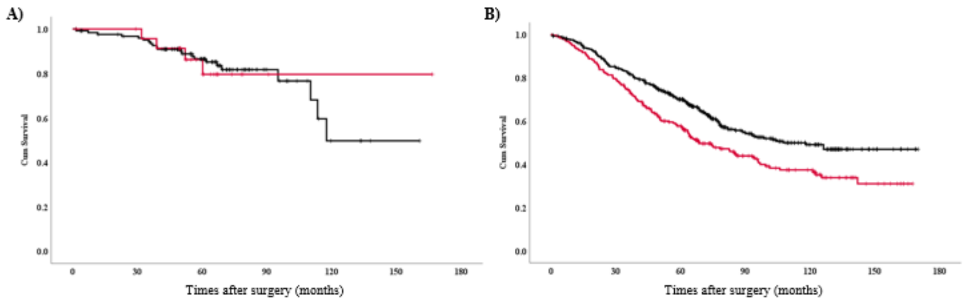

Figure S2. Surgical procedures and outcomes. Overall survival curves stratified by the degree of mediastinal lymph node dissection in thoracoscopic surgery (A) and thoracotomy (B). Black line: lobe-specific lymph node dissection; Red line: systemic lymph node dissection.
